# Microparticles in Pregnancy Complicated by Intrauterine Growth Restriction: A Systematic Review of Literature

**DOI:** 10.3390/medicina62040658

**Published:** 2026-03-30

**Authors:** Nikoleta Aikaterini Xixi, Rozeta Sokou, Fotios Grigoropoulos, Georgia Gkaroutsou, Paraskevi Volaki, Styliani Paliatsiou, Zoi Iliodromiti, Anastasios G. Kriebardis, Nicoletta Iacovidou, Theodora Boutsikou

**Affiliations:** 1 Neonatal Department, Aretaieio Hospital, School of Medicine, National and Kapodistrian University of Athens, 11528 Athens, Greece; nerinaxixi@med.uoa.gr (N.A.X.); rosesok@med.uoa.gr (R.S.); fotis.grigoro@gmail.com (F.G.); gdgkaroutsou@gmail.com (G.G.); voulavolaki@med.uoa.gr (P.V.); stpaliatsiou@med.uoa.gr (S.P.); ziliodromiti@med.uoa.gr (Z.I.); niakobid@med.uoa.gr (N.I.); 2 Laboratory of Reliability and Quality Control in Laboratory Hematology (HemQcR), Department of Bio-Medical Sciences, School of Health & Caring Sciences, University of West Attica (UniWA), 12243 Athens, Greece; akrieb@uniwa.gr

**Keywords:** cell-derived microparticles, extracellular vesicles, fetal growth restriction, biomarkers, preeclampsia

## Abstract

*Background and Objectives*: Intrauterine growth restriction (IUGR) affects 5–10% of pregnancies and is associated with increased perinatal morbidity and long-term complications for the neonate. Extracellular vesicles (EVs), and in particular, microparticles (MPs), have emerged as potential biomarkers of pregnancy complications; however, the literature on their role in neonates remains limited. To investigate the functional characteristics, concertation in maternal blood and potential role of MPs in IUGR. *Materials and Methods*: PubMed, Scopus and preprint servers were systematically searched for studies on MPs in correlation with IUGR from 1 August until 18 September 2025. Data on MP characteristics and concentration in maternal blood samples in the context of IUGR were collected. The systematic review is registered in PROSPERO (CRD420251156939). *Results*: A total of 12 studies fulfilled the inclusion criteria and were included in the review. In IUGR-complicated pregnancies, circulating MPs exhibited preserved procoagulant activity despite minimal quantitative differences compared to controls. Platelet-, endothelial-, and placenta-derived MPs were most frequently studied. Clinically, elevated AV+ placenta-derived MPs were associated with increased risk of IUGR, whereas MPs from isolated IUGR pregnancies showed limited predictive value. *Conclusions*: MPs play a crucial role in the pathophysiology of IUGR through their interplay in coagulation, inflammation, and vascular dysfunction. They show potential as predictive biomarkers, particularly in cases of preeclampsia-associated IUGR, reflecting systemic maternal endothelial and inflammatory changes. However, their utility in isolated IUGR appears limited, likely due to the predominantly local placental origin of the pathology.

## 1. Introduction

Intrauterine growth restriction (IUGR) is described as failure of the fetus to reach its genetically predisposed growth potential, characterized by a pathological curve of intrauterine growth [1]. It complicates approximately 5–10% of pregnancies and is directly linked to the fetoplacental unit, with its pathogenesis lying in placental, maternal and fetal factors [2]. IUGR increases both fetal mortality and morbidity and subsequently leads to worse neonatal outcomes, as well as long term complications, including but not limited to cardiovascular disease, metabolic syndrome, asthma and impaired lung function [3].

The prenatal diagnosis of IUGR is based on abnormal umbilical and uterine Doppler findings. Increased resistance indicates impaired flow, whereas absent or reversed end-diastolic flow signals severe placental insufficiency. A reduced middle cerebral artery PI reflects the “brain-sparing” effect, and cardiac or venous Doppler helps assess fetal well-being [4]. Doppler studies are essential for diagnosing and monitoring IUGR but do not allow for early intervention. Diagnosis is further supported by ultrasound-estimated fetal weight falling below the 10th percentile for the corresponding gestational age [5].

The term extracellular vesicles (EVs) is used to describe a heterogenous group of membranous particles that are released by cells through various mechanisms during apoptosis, cellular aging, or following cell stimulation and appear to play a pivotal role in intercellular communication, the disposal of material, and membrane repair [6,7]. Microparticles (MPs) are a subcategory of EVs that are created through outward budding and pinching of the plasmatic membrane. In current nomenclature, the term “microparticles” is often used to describe “large EVs” or “ectosomes”, distinguishing them from exosomes, which originate from the endosomal pathway via the release of multivesicular bodies. They are typically larger than exosomes but smaller than apoptotic bodies, though there is overlap in size and content. Their release by cells can impact a wide array of cellular processes, and changes in their concentration or components have been associated with several pathologies [8].

MPs are typically sized between 0.1–1 µm and are characterized by exposure of phosphatidylserine (PS), which can be detected with Annexin V (AV), along with the presence of cell-specific surface markers such as platelet or endothelial proteins. Although the terms EV and MP are sometimes used synonymously in clinical literature, it is essential to distinguish MPs by their specific biogenesis from the plasma membrane, as opposed to the intracellular origin of smaller vesicles. Detection poses a challenge because their small size and low refractive index (making them hard to distinguish from background noise) cannot be surpassed by most instruments [9]. Flow cytometry (FC) is the most used technique, offering analysis of size, count, and marker expression, but requires careful calibration. Complementary methods such as resistive pulse sensing (RPS) and nanoparticle tracking analysis (NTA) provide concentration and size distribution, whereas ELISA-based assays allow for the quantification of MPs bearing specific surface proteins in bulk samples [10]. Since no single method can fully characterize MPs, reliable identification relies on combining size, surface marker profiling, and phosphatidylserine positivity (AV+) to distinguish them from exosomes or non-vesicular elements [9,10].

Practically, the distinction between these populations is primarily based on their biogenesis. Although EVs are the recommended universal descriptor for all lipid-bilayer-enclosed particles, the term “microparticle” specifically refers to those originating from the outward budding of the cell surface. In accordance with the Minimal Information for Studies of Extracellular Vesicles (MISEV) criteria, the term EV acts as an umbrella classification, whereas MPs represent a distinct functional subset characterized by their plasma-membrane-derived lipid and protein composition [10].

Numerous studies have investigated the role of MPs in pregnancy complications, including PE, GDM, preterm birth and spontaneous abortions. In the case of PE, endothelial activation and vascular dysfunction, a shared pathogenetic mechanism with IUGR, is accompanied by an increased production of MPs of endothelial, platelet derived, trophoblastic and leukocyte origin in women with PE in comparison to healthy pregnant controls as they reflect cellular activation and may contribute to hypercoagulable and inflammatory states, which can lead to the development of PE [11,12]. It is noteworthy that disease-related MPs differ from those of healthy populations not only in total concentration but also in characteristics, as an outcome of the specific process that led to their production (i.e., hypoxia, oxidative stress, etc.) [13]. Although several studies have reported altered concentrations and compositions of MPs in pregnancies complicated by IUGR, and multiple reviews present how total EV differ in these population, no concise review including solely MPs in IUGR during pregnancy exists. In this systematic review, we aimed to examine the current evidence on the concentration, cellular origin, and functional characteristics of MPs in pregnancies complicated by intrauterine growth restriction (IUGR), with a focus on their potential roles in the pathogenesis of this condition, and fetal outcomes.

## 2. Materials and Methods

This systematic review was developed in accordance with the PRISMA (Preferred Reporting Items for Systematic Reviews and Meta-Analyses) guidelines and the full checklist can be found in the Supplementary Material Data [14]. Inclusion criteria, data synthesis methods, and results were predefined in a protocol registered in PROSPERO (CRD420251156939) and are available online [15].

### 2.1. Search Strategy

PubMed, Scopus, preprint servers (namely, medRxiv and Research Square) and the references of the retrieved articles were systematically searched from 1 August until 18 September 2025. The search was initially conducted by one single reviewer (NAX) and checked by a second reviewer (RS).

During the review process, we decided to repeat and expand our literature search for two primary reasons: first, to enhance methodological rigor by transitioning from a single reviewer to a multi-reviewer independent screening process (involving RS and NAX), and second, to update the search period as the initial results were becoming outdated. Hence, all relevant articles in English until 28 February 2026 were retrieved with no geographical or time restrictions. We used pre-specified keywords, along with Boolean operators, to create a search phrase: microvesicles, microparticles, extracellular vesicles, vesicles, intrauterine growth restriction, fetal growth restriction. The relevant amendment (detailing the literature search update) was made publicly available with PROSPERO.

### 2.2. Eligibility Criteria

Two independent investigators (NAX and RS) conducted the literature search. Any discrepancies were discussed with the corresponding author. We assessed for observational studies, case–control studies, randomized controlled trials, case reports and case series, and experimental studies that investigated the characteristics and concentration of MPs measured in maternal blood samples in the context of IUGR. Reviews of any kind, meta-analyses, editorials, letters to the editor and comments were excluded from the study, as well as studies reporting on EVs as a total, without any specific mention of MPs, or studies referring to any other kind of EV apart from MPs (exosomes, apoptotic bodies, etc.).

### 2.3. Data Extraction

For data extraction, collective tables were compiled, which included data on the first author, publication year, country, study type, study duration, study design, study population, and key information regarding the measurement of MPs and their correlation with IUGR.

## 3. Results

**Figure 1:**
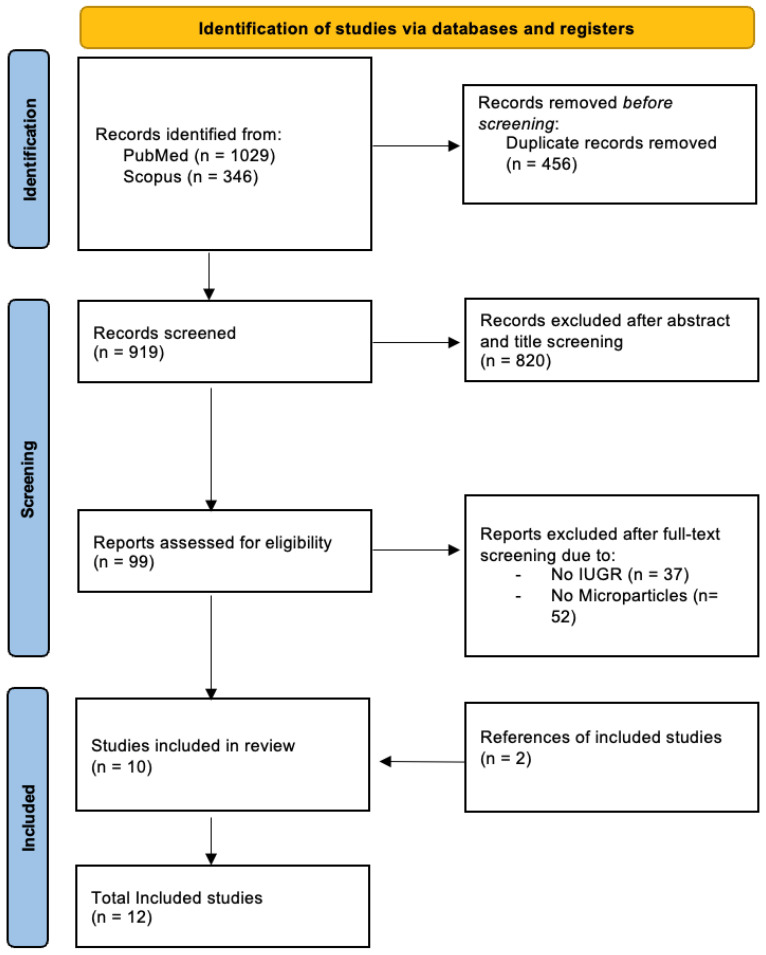
PRISMA Flow Diagram.

Figure 1 shows the flow diagram for study selection. Out of the 1375 initially retrieved articles, 12 met the inclusion criteria and were included in our study [12,16,17,18,19,20,21,22,23,24,25,26]. The majority of the studies were designed as case–control studies, whereas all except for two experimental studies [19,20] recruited human subjects. Six studies defined IUGR as estimated fetal weight (EFW) below the 10th percentile [16,17,18,21,22,23], whereas two used a stricter cut-off (5th percentile) [12,24]. Table 1 summarizes the studies’ characteristics.

### 3.1. MPs Sampling and Detection

Among the studies included in this review different methods for blood collection, processing, and detection were employed. Flow cytometry was the most frequently used technique, applied by 7/11 studies [12,16,17,21,22,23,25]. Blood was generally obtained from the antecubital vein and collected into sodium citrate or buffered sodium citrate vacutainers. Processing typically involved sequential centrifugation steps to prepare platelet-poor or cell-free plasma (PPP and CFP respectively), and plasma samples were then stored at −80 °C until analysis. Table 2 summarizes the sample preparation methods of each of the included studies.

### 3.2. Most Commonly Utilized MP Markers

Platelet-derived MPs were predominantly characterized by CD41+, CD61+, or CD51+ expression [12,16,23,25], whereas endothelial MPs exhibited CD31+, CD144+, or CD62e+, with CD31/CD41 dual-labelling distinguishing endothelial from platelet-derived vesicles [16,21,23,25]. Leukocyte-derived MPs were identified by CD45+, CD4+, or CD8+ expression [16,25], and erythrocyte-derived MPs by glycophorin A+ [25]. Placental or Syncytiotrophoblast-derived MPs were identified by Placental Alkaline Phosphatase (PLAP) +, ED822+, or sFlt-1+ [17,24,25]. Annexin V (AV) was utilized as a general marker for all MP populations independent of origin [12,16,17,22,23,25]. In two studies, in addition to studies investigating endogenous MPs, experimental models with artificial PS/PC vesicles were employed to mimic circulating microvesicles (Table 3) [19,20].

### 3.3. MP Procoagulant Activity in IUGR

Table 3 also summarizes the individual study finding regarding the role of MPs in IUGR. Pregnancy is reported as a condition of increased coagulability, as part of the normal adaptive changes that happen during this life period [12,22]. However, conditions such as IUGR are characterized by hypercoagulability and an increased inflammatory response. Procoagulant MP activity in IUGR has been found to be significantly higher than expected in normal pregnancy (up to 21.26 times higher), suggesting that inflammatory and procoagulant processes promote MP overproduction [18]. Experimental evidence from mouse models confirms that PS MPs cause significant reductions in fetal weight (*p* < 0.01) [19]. In these models, PS-treated mice exhibited significantly elevated thrombin–antithrombin (TAT) levels compared to controls (22.7 ± 15.7 mg/L vs. 6.6 ± 3.6 mg/L, *p* < 0.05), with reduced platelet counts (88.2 ± 31.0 × 10^10^/L vs. 112.0 ± 21.0 × 10^10^/L, *p* < 0.05) and lower antithrombin activity, indicating increased procoagulant activity in vivo [19,20].

In clinical settings, studies exhibit various findings regarding MP concentrations, but functional procoagulant activity remains a consistent feature. Although some studies reported a trend toward lower leukocyte- and endothelial-derived MPs in isolated IUGR, potentially reflecting increased local consumption or vascular binding linked to endothelial dysfunction [16], others pointed out that total and platelet-derived MPs were decreased in complicated pregnancies, even while their global procoagulant activity remained stable [12]. Notably, most circulating MPs in IUGR may initially be AV, CD14, CD45, CD235a or tissue factor negative, suggesting low detectable PS. However, MPs in these cases keep their procoagulant activity in thrombin generation assays that is abolished by AV, confirming the functional presence of PS. CD31+ (endothelial), CD41+ (platelet), and CD31−/CD41− MPs all exhibited preserved procoagulant function, despite no significant quantitative differences compared to controls [23]. Chen et al. also attributed the hypercoagulable state during PE/IUGR pregnancies to the high concentration of AV+-placenta derived MPs [17].

### 3.4. Predictive Value of MPs

The predictive capacity of circulating MPs is highly dependent on gestational week and the pregnancy outcomes (Table 3). During the first trimester (10–14 weeks), total AV+ MPs show limited reliability as a predictor for isolated IUGR (AUC 0.487), but their predictive power increases when IUGR is associated with PE (AUC 0.82), with research showing a high negative predictive value of 95.3% [21].

In the second trimester (18–23 weeks), although CD62e+ EMPs are significantly elevated in PE-associated IUGR, they demonstrate low isolated predictive value for IUGR (AUC 0.583, sensitivity 61%, specificity 58.1%) [17]. Similarly, the MORE PrePARd study found that at 19–21 weeks, maternal STBM concentrations were not effective for prediction, as in a group of 73 high-risk women, the ROC curve for mid-gestational syncitiotrophoblastic MPs stayed very close to the line of no-discrimination, meaning the marker failed to predict, with enough certainty, PE or other secondary issues such as IUGR and placental abruption at that stage [26]. By the third trimester (27–38 weeks), specific concentrations of AV+ placental MPs (>6524/μL) in severe PE serve as independent predictors for the development of IUGR or fetal distress, as shown by Chen et al. [17]. Interestingly, MP parameters did not correlate with the birth weight/expected birth weight ratio [12].

## 4. Discussion

Our study, incorporating 12 studies on the role of MPs in pregnancy complications, showed that MPs contribute to the pathogenesis of IUGR, whereas their levels and activity differ from healthy controls. Additionally, our review revealed their potential as predictive biomarkers, especially in PE-associated IUGR, although their utility in isolated IUGR remains limited.

MPs offer a significant contribution to coagulation, inflammation and vascular dysfunction, with their role expanding to fetomaternal cross-talk, making them key factors in the pathophysiology of IUGR [27]. During pregnancy, various physiological adaptive changes happen to promote fetal development while also protecting maternal health. Pregnancy itself is a prothrombotic state, with increased procoagulant activity in order to reduce bleeding risk. MPs are crucial mediators in the prothrombotic state of pregnancy, contributing to the initiation of coagulation and thrombus formation. In normal pregnancy, MPs demonstrate increased procoagulant activity in comparison to non-pregnant women [28].

During pregnancy, EVs (including MPs) are released by the placenta, the trophoblast and the fetus itself, ensuring successful implantation, angiogenesis, immune balance, and adequate energy supply for the fetus, contributing to the interplay between maternal and fetal tissues. During pregnancy, EV concentration appears to be higher than in non-pregnant controls, even from the first trimester, and there seems to be an upward trend as the pregnancy progresses [29]. By the second trimester, concentrations rise noticeably, reflecting increasing placental mass and activity, as well as contributions from activated maternal cells such as platelets and endothelial cells. In the third trimester, EV levels reach their highest concentrations and are further elevated in pathological pregnancies such as preeclampsia (PE), gestational diabetes (GDM), and IUGR [30,31]. EVs are responsible for maternal–fetal immunoregulation by preserving a balance between pro- and anti-inflammatory cytokines [32]. Additionally, their cargo consists of factors such as HLA-G, VEGF and miRNAs, all of which promote angiogenesis, placentation and nutrient exchange [29,32].

Specifically, MPs, as byproducts of cell activation or apoptosis from platelets, endothelial cells and monocytes (among others) have an active role in the pathogenesis of vascular dysfunction. They contribute significantly to oxidative stress, as they carry enzymes and proteins that promote the production of reactive oxygen species (ROS), worsening endothelial dysfunction. At the same time, MPs increase the inflammatory response through the activation of proinflammatory cytokines and the adhesion of monocytes to the endothelium. Additionally, endothelial MPs can act as markers of endothelial damage while also being involved in vascular dysfunction by impairing vasodilatory capacity and interacting with the vascular wall [8].

### 4.1. Platelet-Derived MPs

These vesicles have been reported to possess both procoagulant and anticoagulant activities due to the negative charge derived from the exposed membrane. Platelet-derived microparticles exhibit a dual functional profile in hemostasis, encompassing both procoagulant and anticoagulant activities. Their potent procoagulant capacity is primarily attributed to the externalization of phosphatidylserine, which provides a catalytic surface for the assembly of coagulation enzyme complexes, thereby markedly enhancing thrombin generation and fibrin formation, reported to be 50–100 times greater than that of activated platelets [33]. This activity promotes platelet deposition, fibrin network development, and thrombus propagation, contributing to the pathophysiology of thrombotic and cardiovascular disorders. Conversely, certain PMP subpopulations demonstrate anticoagulant and fibrinolytic properties by facilitating plasmin generation and, under certain conditions, inhibiting platelet aggregation, thus modulating clot dissolution and restraining excessive thrombosis [34]. Nevertheless, the precise role of MPs remains poorly understood, and their formation under conditions that replicate in vivo hemostasis, and thrombosis has yet to be clearly demonstrated [35].

In IUGR and PE, platelet-derived MPs (marked by CD41 positivity) are increased, indicating platelet activation. These MPs promote the formation of microthrombi in placental vessels, subsequently restricting blood supply to the fetus. By exposing PS, platelet MPs increase coagulation and vascular injury/dysfunction [17]. Interestingly, some studies have suggested that in IUGR, a trend toward lower numbers of platelet-derived MPs has also been observed, which may be explained by their consumption during excessive clotting reactions in the placental microvasculature, adhesion to monocytes and endothelial cells, or sequestration within placental tissue. Despite these lower circulating numbers, platelet MPs maintain their procoagulant activity, supporting thrombin generation and clot formation, which, in turn, causes disturbance in the fetomaternal circulation and IUGR [12,16,19,20].

### 4.2. Placental MPs

The placenta is a major source of MPs, especially STBMs, with studies still showing controversial results regarding their concentrations and pathogenicity. STBMs are by nature procoagulant due to the high TF expression and exposure of negatively charged phospholipids during trophoblast differentiation, a condition that promotes MP shedding and is increased during PE-related changes [28]. In PE, these MPs correlate with maternal endothelial dysfunction, as mentioned in the study by Chen et al., where MPs correlated with proteinuria and need for antihypertensive therapy, highlighting their systemic effects beyond the placenta [17]. Placental hypoxia and oxidative stress promote trophoblast apoptosis and STBM shedding, which exposes procoagulant surfaces and triggers microthrombosis in the placental circulation [18,20]. This reduces uteroplacental blood flow, limiting oxygen and nutrient provision to the fetus, and creates a repeating cycle of ischemia, MP release and coagulation, worsening placental insufficiency and, subsequently, IUGR [17,18].

Interestingly, STBMs are elevated in PE but not in normotensive IUGR, as indicated in the study by Goswami et al. This suggests that, although systemic maternal mechanisms contribute to the maternal syndrome in PE, isolated IUGR primarily reflects local placental pathology, as proposed also by Bretelle et al., where it was suggested that the effects on the vasculature are mainly limited to the feto–placental unit, consistent with IUGR being primarily a placental pathology rather than a systemic maternal syndrome. This distinction may explain why normotensive IUGR patients often exhibit restricted fetal growth without the systemic endothelial and inflammatory changes observed in PE [12,17,24].

### 4.3. Endothelial MPs

On the other hand, endothelial-derived MPs contribute to the hypercoagulable state and vascular dysfunction by promoting maternal endothelial injury and thrombosis [16,17]. As already established, endothelial dysfunction is a hallmark of placental insufficiency in IUGR [35]. According to Alijotes-Reig et al., elevated levels of endothelial-derived MPs (CD144+, CD31+/CD41−) have been observed in IUGR-affected pregnancies, reflecting endothelial activation or injury. Thus, endothelial cell activation can be detected as circulating endothelial MPs, which themselves cause endothelial dysfunction and an increased inflammatory response. Additionally, circulating MPs activate the maternal endothelium and promote microthrombi formation, further limiting placental blood flow [12,16,20,21,22,23,25].

As previously mentioned, pregnancy is a state of continuous adaptive changes, with many dynamic changes occurring throughout its course [28,36]. Leukocyte-derived MPs (CD45+) reflect systemic immune activation and the initiation of inflammatory responses in IUGR [16,24]. Placental ischemia and hypoxia trigger the release of MPs from stressed trophoblasts, endothelial cells, platelets, and leukocytes. These MPs carry cytokines and adhesion molecules that exacerbate endothelial dysfunction, creating an environment in which hypoxia and MP release reinforce one another, which further promote IUGR [18,23]. Activated leukocytes cause systemic and local inflammation, which causes more damage to endothelial cells and worsens placental perfusion. Goswami at al. also implicated that, in PE, excess STBMs stimulate innate immune cells, worsening maternal inflammatory responses, whereas in normotensive IUGR, limited STBM release results in minimal maternal immune activation [24].

### 4.4. Predictive Value

Extracellular vesicles have been proposed as possible biomarkers in various physiological and pathological conditions due to their stability in body fluids, ability for early detection (before symptoms even arise), and disease-specific molecular content, acting as molecular “snapshots” of their parental cells [37]. Emerging evidence suggests that epigenetic biomarkers, including EVs and MPs, could play a dual role in managing thrombophilia-related pregnancy complications. First, in risk stratification, by identifying women at high risk for conditions such as preeclampsia, IUGR, or recurrent pregnancy loss and second, as potential therapeutic targets [38]. Other studies suggest that EVs derived from mesenchymal stem cells or umbilical cord blood may affect treatment in pregnancies complicated by IUGR. These EVs appear capable of improving fetal growth, reducing inflammation, and enhancing placental and fetal vascular function. Their cargo, including specific microRNAs such as miR-150, may regulate angiogenesis and placental development, indicating further that EVs could serve not only as biomarkers but also as potential therapeutic agents to modulate placental function and support fetal development [39].

Our review included three studies that investigated the potential predictive value of MPs in IUGR [17,21,22]. All three suggested that MPs may be useful predictive biomarkers in pregnancy complications, particularly in PE associated with IUGR. Chen et al. demonstrated that elevated levels of AV+ placental extracellular vesicles were strongly associated with subsequent IUGR and FD in severe PE, showing high sensitivity, specificity, and temporal predictive value. Interestingly, MP levels were negatively correlated with the time that took to develop IUGR in both PE/IUGR and isolated IUGR groups [17]. However, a consistent finding across these studies is that MPs exhibit limited predictive value for isolated IUGR. Generally, women who developed normotensive IUGR did not show significant changes in circulating MP levels compared to controls. As previously mentioned, normotensive IUGR reflects local placental dysfunction, which, compared with the already presented predictive value for PE and PE/IUGR, indicates that MPs are effective predictive markers for PE and PE complicated by IUGR because they reflect maternal systemic endothelial and inflammatory changes. However, they have low predictive value for isolated IUGR, which is largely confined to the placenta [21,22].

### 4.5. MP Discrepancies Across the Included Studies

The discrepancies in MP concentrations reported across the studies, specifically regarding STBM and PMP levels, are likely due to significant variations in study design and methodology. A primary factor is the gestational age at sampling, which ranges from early screening at 10–14 weeks to late in the third trimester [17,21]. Since it has been shown that EV concentrations naturally increase as pregnancy progresses, results are highly dependent on the sampling window [29,30,40,41]. Furthermore, methodological differences in MP detection, such as varying centrifugation protocols and the lack of standardized flow cytometry settings, impact absolute counts and sensitivity [9,10]. Finally, the markers used to define MP subsets (e.g., PLAP vs. sFlt-1 for placental origin) and the clinical heterogeneity of the cohorts, specifically the distinction between isolated IUGR and PE-associated IUGR, further explain the differing results, as according to several studies, maternal systemic factors in PE drive a more robust MP release compared to localized placental pathology [12,17,24].

### 4.6. Limitations

Our study has limitations. Firstly, most studies were observational or case–control in nature, limiting the evaluation of confounding factors regarding MPs in the pathogenesis of IUGR. Secondly, the majority of research focused on PE-associated IUGR, with relatively few studies investigating isolated, normotensive IUGR, restricting the ability to generalize our findings to all IUGR cases, regardless of pathogenetic mechanism. Additionally, methodological variability in MP characterization, may have contributed to inconsistent measurements and interpretations, highlighting the need for the standardization of MP detection methods. Finally, although MPs show potential as predictive biomarkers, the absence of longitudinal, large-scale prospective studies limits the ability to establish cut-off values or timing for clinical application.

## 5. Conclusions

MPs are involved in key mechanisms in the pathogenesis of IUGR, influencing coagulation, vascular function, and maternal–fetal communication. Their value as predictive biomarkers, especially in PE/IUGR, is promising, whereas their utility in isolated IUGR remains limited due to the primarily placental origin of the pathology. More studies are necessary to standardize methods for MP detection and characterization, to make it easier to identify their cellular origins, and to shed light on their roles in IUGR. Ultimately, the clinical integration of MP profiling into future diagnostic and screening algorithms offers a potential strategy for the early identification of high-risk pregnancies. Specifically, a multi-modal approach combining maternal uterine artery Doppler with a specific panel of MPs could enhance the sensitivity of screening for IUGR as early as the first trimester. Longitudinal and large-scale cohort studies could establish clinically relevant thresholds for predictive use, whereas experimental studies may identify targeted interventions to modulate MP activity and improve fetal outcomes. Furthermore, longitudinal MP monitoring could serve as a clinical tool to help determine the optimal window for delivery in severe growth restriction cases.

## Figures and Tables

**Table 1:** Individual Study Characteristics.

First Author	Year	Population, N	IUGR Definition	Study Type	Country	Time Period	Aim
Alijotas-Reig et al. [16]	2012	70 PE 70 isolated IUGR	EFW < 10th p. on U/S and Doppler PI in the UA above the 95th p., or EFD < 3rd p. irrespective of the UA Doppler.	Case–control study	Spain	2008–2009	To investigate the behavior of circulating cMPs in sPE and IUGR.
Bretelle et al. [12]	2003	24 PE and IUGR 18 IUGR 15 normal pregnant women	EFW < 5th p. on U/S, and BW < 5th p.	Case–control study	France	NA	To analyze the level and procoagulant activity of MPs in normal pregnancy and PE or IUGR-complicated pregnancies.
Chen et al. [17]	2025	80 sPE patients 41 pregnant controls 27 non-pregnant women controls	EFW < 10th p. for the GA measured using obstetric U/S.	Single-center retrospective case–control study	China	2018–2019	To assess whether plasma levels of pEVs, lactadherin, and prothrombotic markers predict IUGR and/or fetal distress in sPE.
Fitzgerald [26]	2012	16 PE patients 56 pregnant controls	NA	Preliminary multicenter prospective cohort study		NA	To evaluate whether syncytiotrophoblastic MPs can serve as an accessory marker to conventional Doppler sonography to better identify pregnant women who will develop PE.
Goswami et al. [24]	2006	15 EOPE 10 LOPE 10 nIUGR 35 normal pregnant women	EFW or AC < 5th p. on U/S	Prospective case–control study	UK	NA	To examine whether the shedding of STBM in nIUGR occurs to the same extent as in PE.
Jadli et al. [21]	2019	41 PE 68 IUGR 16 isolated PE 25 PE and IUGR	EFW < 10th p. on U/S, and BW < 10th p.	Case–control study	India	2014–2017	To investigate whether combining CD62e, CPP, and PlGF levels at mid-pregnancy can help predict PE in first-time mothers.
Jadli et al. [22]	2017	112 controls 16 isolated PE 17 PE complicated by IUGR	EFW < 10th p. on U/S, and BW < 10th p.	Case–control study	India	2014–2016	To investigate the role of CPP, PlGF and cMPs, alone and in combination, for the prediction of PE at 10–14 GW.
Lok et al. [25]	2008	20 PE 20 normal pregnancies 20 non-pregnant women	NA	Case–control study	Netherlands	NA	To investigate whether soluble Flt-1 is associated with plasma MPs in PE, to assess if the number of Flt-1–bearing MPs is higher in PE compared to normal pregnancy, to identify the cellular origin of these MPs, and to determine whether full-length or truncated forms of Flt-1 are present on circulating MPs.
Makris et al. [18]	2015	One 33-year-old, 37th GW	EFW < 10th p. on U/S	Case study	Greece	NA	To present the potential correlation between a very high potency of MPs and IUGR.
Omatsu et al. [20]	2005	82 PS/PC mice 99 normal mice	NA	Experimental interventional animal study	Japan	NA	To investigate the role of PS/PC in the pathophysiology of PE.
Salomon et al. [23]	2009	262 healthy nulliparous women	EFW < 10th p. on U/S	Prospective cohort study	Israel	NA	To determine whether plasma levels of MPs measured at 24 GW could predict pregnancy complications.
Sugimura et al. [19]	1999	mice	NA	Experimental interventional animal study	Japan	NA	To investigate the role of PS derived from activated platelets in the placental circulation.

N, Number; NA, Not Applicable; IUGR, Intrauterine Growth Restriction; sPE, Severe Preeclampsia; EFW, Estimated Fetal Weight, p; Percentile, GA, Gestational Age; U/S, Ultrasound; BW, Birth Weight; pEVs, Placental Extracellular Vesicles; CPP, Copeptin; PIGF, Placental Growth Factor; MPs, Microparticles; PI, Pulsality Index; UA, Umbilical Artery; EOPE, Early Onset Preeclampsia; LOPE, Late Onset Preeclampsia; nIUGR, Normotensive Intrauterine Growth Restriction; AC, Abdominal Circumference; STBM, Syncytiotrophoblast Microparticles; PS, Phosphatidylserine; PC, Phosphatidylcholine; Flt-1, Fms-Like Tyrosine Kinase 1.

**Table 2:** Sample preparation methods.

First Author	MP Detection	Blood Sampling					
		MP Source		Quantity	Tube Type	CF Method	Storage
Alijotas-Reig et al. [16]	Flow cytometry	Venous Blood	Antecubital vein	10 mL	sodium citrated vacutainers	Step 1: 1500× *g* for 15 min Step 2: 13,000× *g* for 2 min → PPP	−80 °C
Bretelle et al. [12]	Flow cytometry	Whole blood	NA	NA	Sodium citrated vacutainers	Step 1: 1500× *g* for 15 min Step 2: 13,000× *g* for 2 min → PPP	NA
Chen et al. [17]	Flow cytometry	Venous Blood	Antecubital vein	5 mL	0.32% sodium citrate vacutainer tubes	Step 1: 120× *g* for 20 min at room temperature → PRP Step 2: 1500× *g* for 20 min at room temperature → PPP Step 3: 13,000× *g* for 2 min → CFP	NA
Fitzgerald et al. [26]	NA	NA	NA	NA	NA	NA	NA
Goswami et al. [24]	ELISA	Venous Blood	Antecubital vein	5 mL	NA	High speed centrifugation	−80 °C
Jadli et al. [22]	Flow cytometry-FACS	Venous blood	NA	5 mL	Trisodium citrated vacutainers	1500× *g* for 15 min at room temperature → PPP	−80 °C
Jadli et al. [21]	Flow cytometry-FACS	Venous blood	NA	5 mL	Trisodium citrated vacutainers	1500× *g* for 15 min at room temperature → PPP	−80 °C
Lok et al. [25]	Flow cytometry	Venous Blood	Antecubital vein	9 mL	Buffered sodium citrate vacutainers	20 min at 1560× *g* and 20 °C → CFP	−80 °C
Makris et al. [18]	NA	Whole blood	NA	NA	NA	NA	NA
Omatsu et al. [20]	NA	NA	NA	NA	NA	NA	NA
Salomon et al. [23]	Flow cytometry	Venous Blood	Antecubital vein		3.2% buffered sodium citrate vacutainers	Step 1: 1500× *g* for 10 min at room temperature Step 2: 13,000× *g* for 3 min → PPP	−80 °C
Sugimura et al. [19]	ΝA	NA	NA	NA	NA	NA	NA

MP, Microparticle; mL, milliliters; FACS, Fluorescence-activated cell sorting; ELISA, enzyme-linked immunosorbent assay; min, minutes; PRP, Platelet Rich Plasma; PPP, Platelet Poor Plasma; CFP, Cell-Free Plasma; NA, Not Applicable.

**Table 3:** MP characteristics.

First Author	Type, Cell of Origin	MP Markers	Correlation
Alijotas-Reig et al. [16]	Platelet-derived, leukocytic, endothelial cells, trophoblastic, erythrocytic	AV+, CD41+, CD31-/CD41−, CD45+, CD31+/CD41−, CD144+	No significant differences in isolated IUGR vs. controls.
Bretelle et al. [12]	Platelet-derived, endothelial cells	CD41+, CD51+, AV+	Trend toward lower MPs in IUGR, but unchanged procoagulant activity
Chen et al. [17]	Syncytiotrophoblast-derived	AV+ and PLAP+	Elevated AV+ pcMPs predicted IUGR/FD with high sensitivity/specificity.
Fitzgerald et al. [26]	Syncytiotrophoblast-derived	NA	Preliminary results showed that maternal syncitiotrophonlastic MP concentration at mid-gestation does not predict the development of PE or associated pregnancy pathologies (namely IUGR, intrauterine fetal demise, placental abruption, and premature delivery).
Goswami et al. [24]	Syncytiotrophoblast-derived	PLAP+	Increased STBM only in EOPE. Levels in LOPE and in IUGR similar to controls.
Jadli et al. [21]	Endothelial	CD62e+	Biomarkers (including elevated CD62e+ MPs) specific for PE ± IUGR; not predictive for isolated IUGR.
Jadli et al. [22]	Circulating	AV+	Biomarkers (including AV+ MPs) showed poor predictive value for isolated IUGR; predictive only for PE ± IUGR.
Lok et al. [25]	Platelet-derived, placental, endothelial, erythrocytic	AV, CD61, CD62e, CD66b, Glycophorin, CD4, CD8, sFlt-1, ED822	No correlation between BW and Flt-1-exposing MPs
Makris et al. [18]	NA	NA	Strongly elevated MP activity linked to IUGR.
Omatsu et al. [20]	NA	PS/PC+	PS/PC group showed reduced fetal/placental weight → IUGR phenotype.
Salomon et al. [23]	Endothelial cells, platelet-derived	CD31+, CD41+, AV+	CD31+, CD41+ MPs not different in IUGR vs. uncomplicated pregnancy.
Sugimura et al. [19]	ΝA	PS/PC+	Artificial PS/PC vesicles induced IUGR phenotype in mice.

NA, Not Applicable; AV, Annexin V; PLAP, Placental Alkaline Phosphatase; PS/PC, Phosphatidylserine/Phosphatidylcholine; sFlt-1, Fms-like tyrosine kinase-1.

## Data Availability

No new data were created or analyzed in this study.

## Supplementary Materials

The following supporting information can be downloaded at: https://www.mdpi.com/article/10.3390/medicina62040658/s1, Table S1: PRISMA 2020 checklist.
